# Association between the risks of contrast-induced nephropathy after diagnostic or interventional coronary management and the transradial and transfemoral access approaches

**DOI:** 10.34172/jcvtr.2020.08

**Published:** 2020-02-12

**Authors:** Ata Firouzi, Mohammad Javad Alemzadeh-Ansari, Naser Mohammadhadi, Mohammad Mehdi Peighambari, Ali Zahedmehr, Bahram Mohebbi, Reza Kiani, Hamid Reza Sanati, Farshad Shakerian, Alireza Rashidinejad, Behshid Ghadrdoost, Raana Asghari, Simin Shokrollahi Yancheshmeh

**Affiliations:** Cardiovascular Intervention Research Center, Rajaie Cardiovascular Medical and Research Center, Iran University of Medical Sciences, Tehran, Iran

**Keywords:** Contrast-Induced Nephropathy, Transfemoral Access, Transradial Access, Percutaneous Coronary Intervention

## Abstract

***Introduction:*** The risk of contrast-induced nephropathy (CIN) as a common and important complication of coronary procedures may be influenced by the vascular access site. We compared the risks of CIN in diagnostic or interventional coronary management between patients treated via the transradial access (TRA) and those treated via the transfemoral access (TFA).

*** Methods:*** Patients undergoing invasive coronary catheterization or percutaneous coronary intervention (PCI) were enrolled. We excluded patients with congenital or structural heart disease and those with end-stage renal disease on dialysis. Based on the vascular access site used for invasive coronary catheterization, the patients were divided into 2 study groups: the TFA and the TRA. CIN was defined as an absolute (≥0.5 mg/dL) or relative (>25%) increase in the baseline serum creatinine level within 48 hours following cardiac catheterization or PCI.

*** Results:*** Overall, 410 patients (mean age = 61.3 ± 10.8 years) underwent diagnostic or interventional coronary management: 258 were treated via the TFA approach and 152 via the TRA approach. The patients treated via the TFA had a significantly higher incidence of postprocedural CIN (15.1% vs 6.6%; *P*= 0.01). The multivariate analysis showed that the TFA was the independent predictor of CIN (OR: 2.37, 95% CI: 1.11 to 5.10, and *P*= 0.027). Moreover, the BARC (Bleeding Academic Research Consortium) and Mehran scores were the other independent predictors of CIN in our study.

*** Conclusion:*** The risk of CIN was lower with the TRA, and the TFA was the independent predictor of CIN after the diagnostic or interventional coronary management.

## Introduction

Contrast-induced nephropathy (CIN) is a common and important complication of procedures that are likely to use contrast media and is associated with increased morbidity and mortality. In spite of improvements in the chemical structures of contrast media, CIN is known as the third cause of hospital-acquired acute kidney injury, increasing both the short- and long-term risks of adverse events such as the need for hemodialysis, myocardial infarction, congestive heart failure, stroke, and death.^1-4^ The reported incidence of CIN after coronary angiography or percutaneous coronary intervention (PCI) varies widely, from 2% to 25%, depending on the baseline characteristics of the patients and the CIN definition in the studies.^5-7^ The current evidence shows that a combination of direct toxic effects on tubular epithelial cells and renal ischemia play a pathogenetic role in the presentation of CIN following procedures,^8-11^ although the exact mechanisms leading to the development of CIN remain unclear. The most important patient-related risk factors for developing postprocedural CIN are diabetes and preexisting renal insufficiency.^2^ Mehran et al developed a simple risk score for predicting CIN after PCI to assess the cumulative risk of the aforementioned risk factors.^12^

Recently, some studies have evaluated 2 routine accesses in angiography and angioplasty and suggested that the transradial access (TRA) may be associated with lower rates of CIN than the transfemoral access (TFA).^13-16^ In contrast, some other reports have not been able to show the superiority of the TRA for the reduction of postprocedural CIN—even in high-volume radial centers.^17,18^ Moreover, there is evidence indicating that different populations may have different risk factors for the development of nephropathy.^19^

Given the dearth of relevant data in our region, we sought to evaluate the association between the use of the 2 vascular access sites of the TRA and the TFA and the presence of CIN in patients undergoing diagnostic or interventional coronary management.

## Materials and Methods

From September 2016 to April 2017, we enrolled all patients aged 18 years or older who were admitted to our tertiary academic medical center for elective or early invasive coronary catheterization or PCI. We excluded patients with congenital or structural heart disease and those with end-stage renal disease on dialysis. Based on the vascular access site used for invasive coronary catheterization, the patients were divided into 2 study groups: the TFA and the TRA.

For a better evaluation of the presence of CIN and accesses, the clinical and procedural characteristics of the patients were compared between the 2 groups. These variables included traditional risk factors, hypotension (a systolic blood pressure <80 mm Hg), the use of the intra-aortic balloon pump, the left ventricular ejection fraction, anemia (hemoglobin <12 mg/dL), hematocrit, the contrast volume, the procedure type (angiography vs PCI), serum creatinine, and the estimated glomerular filtration rate (eGFR). Additionally, at 48 hours post-procedurally, the changes in the levels of serum creatinine and hematocrit were evaluated. None of the patients received any preventive medication for CIN, except 500 mL of normal saline (0.9% sodium chloride), pre- and post-procedurally for up to 12 hours. CIN was defined as an absolute (≥0.5 mg/dL) or relative (>25%) rise in the baseline serum creatinine level within 48 hours post cardiac catheterization or PCI.

Evidence shows that periprocedural bleeding is significantly associated with CIN, and there is a correlation between the incidence of CIN and the severity of bleeding.^15^ Thus, we employed the Bleeding Academic Research Consortium (BARC) score for the evaluation of bleeding severity in our patients.^20^ We also drew upon the Mehran risk score, which is a simple risk score for the assessment of individual patient risk stratification for the development of postprocedural CIN.^12^

### Statistical Analysis

The statistical analyses were performed with the SPSS software, version 11.0 for Windows (SPSS Inc, Chicago, Illinois). Mean standard deviations (SDs) and frequencies were used for the descriptive analyses. For the evaluation of the distribution of the data, the one-sample Kolmogorov–Smirnov test was utilized. The mean variables between the 2 groups were compared using the independent *t*-test or the Mann–Whitney U test. The qualitative data were compared using the χ^2^ test. Multivariable logistic regression models were applied to identify the associates of CIN following the procedures. A multivariable logistic regression model via the backward variable selection method was constructed based on the variables that yielded statistical significance in the univariable logistic regression.

## Results

During the study period, 410 patients—comprised of 289 men and 121 women—at a mean age of 61.3±10.8 years underwent diagnostic or interventional coronary management. Of the 410 patients, 258 were treated via the TFA and 152 via the TRA. The baseline and clinical characteristics of the patients, categorized by access site, are depicted in Table 1. The patients treated with the TFA approach had significantly higher BARC and Mehran scores at baseline than did the TRA group; nonetheless, the differences between the 2 groups with respect to the other baseline characteristics did not constitute statistical significance (Table 1).

**Table 1:** Comparisons of the baseline and clinical characteristics between the femoral and radial access sites

	**TFA (n=258)**	**TRA (n=152)**	**Total (n=410)**	***P***
Age (y) (%)	61.9 ± 11.3	60.14 ± 9.9	61.30 ± 10.85	0.06
Age (y) (%) <75	227 (88%)	138 (90.8%)	365 (59%)	0.38
≥75	31 (12%)	14 (9.2%)	45 (11%)	
Sex (male)	176 (68.2%)	113 (74.3%)	289 (70.5%)	0.18
BMI (kg/m^2^)	26.5 ± 4.3	26.6 ± 5.3	26.60 ± 4.74	0.65
Smoking (%)	62 (24%)	42 (27.6%)	104 (25.4%)	0.41
Hypertension (%)	114 (44.2%)	60 (39.5%)	174 (42.4%)	0.35
Dyslipidemia (%)	35 (13.6%)	18 (11.8%)	53 (12.9%)	0.61
Diabetes mellitus (%)	79 (30.9%)	54 (36.2%)	133 (32.4%)	0.26
LVEF (%)	41.7 ± 10.02	43.35 ± 10.46	42.31 ± 10.20	0.055
Hypotension (%)	3 (1.2%)	0(0)	3 (0.7%)	0.18
Contrast (%)				0.07
Visipaque	103 (39.9%)	44 (28.9%)	147 (35.9%)	
Ultravist	122 (47.3%)	82 (53.9%)	204 (49.8%)	
Omnipaque	33 (12.8%)	26 (17.1%)	59 (14.4%)	
SCr before (mg/dL )	1.14 ± 0.3	1.10 ± 0.2	1.13 ± 0.30	0.43
SCr after (mg/dL )	1.19 ± 0.3	1.09 ± 0.2	1.15 ± 0.35	0.05
∆ SCr	0.04 ± 0.2	0.00 ± 0.2	0.02 ± 0.25	0.01
eGFR	64 ± 21.07	66.87 ± 19.4	65.06 ± 20.52	0.12
Contrast volume (mL)	200.7 ± 123.4	163.9 ± 107.2	186.37 ± 119.08	<0.0001
Hct before (mg/dL)	39.78 ± 5.33	39.23 ± 6.42	39.58 ± 5.76	0.88
Hct after (mg/dL)	37.11 ± 5.52	39.91 ± 2.95	38.15 ± 15.25	0.08
∆ Hct	2.97 ± 3.20	2.39 ± 3.03	2.76 ± 3.15	0.01
BARC score				0.02
low (0,1)	207(80.2)	135(88.8%)	342 (83.4%)	
High (≥2)	51(19.8)	17(11.2%)	68 (16.6%)	
Mehran score	96 (37.2%)	70 (46.1%)	166 (40.5%)	0.04
low (<6)				
Intermediate (6-10)	121 (46.9%)	57 (37.5%)	178 (43.4%)	
High (11-16)	35 (13.6%)	25 (16.4%)	60 (14.6%)	
Very high (>16)	6 (2.3%)	0(0)	6 (1.5%)	
V/ GFR	6.36 ± 4.42	2.96 ± 4.90	5.10 ± 35.38	<0.0001
V/ BMI	7.54 ± 4.99	5.86 ± 4.01	6.92 ± 4.72	<0.0001
CIN	39 (15.1%)	10 (6.6%)	49 (12%)	0.01

LVEF, left ventricular ejection fraction; BARC, Bleeding Academic Research Consortium; GFR, glomerular filtration rate; CIN, contrast-induced nephropathy; BMI, body mass index; Hct, hematocrit; SCr, serum creatinine; TFA: trans-femoral access; TRA: trans-radial access.

CIN occurred in 49 (12%) patients, with the primary end point being defined as an absolute (≥0.5 mg/dL) or relative (>25%) increase in the baseline serum creatinine level. The patients treated with the TFA approach had a significantly higher incidence rate of postprocedural CIN than did those treated with the TRA approach (15.1% vs 6.6%; *P*=0.01). Although the volume of the contrast medium used during the procedure was higher in the TFA approach than in the TRA approach, the multivariable regression analysis demonstrated that the volume of the contrast medium was not an independent variable for the development of postprocedural CIN (*P* = 0.341) (Table 2). After the multivariate analysis, the independent predictors of CIN were the BARC score (OR: 5.40, 95% CI: 2.07 to 14.09, and *P* = 0.001), the Mehran score (OR: 1.88, 95% CI: 1.22 to 2.91, and *P* = 0.004), and the TFA (OR: 2.37 95% CI: 1.11 to 5.10, and *P* = 0.027) (Table 2).

**Table 2:** Multivariable regression analysis between CIN and some variables

	**Beta**	**OR (95% CI)**	***P***
BARC score	1.68	5.40 (2.07-14.09)	0.001
Femoral access	0.86	2.37 (1.11-5.10)	0.027
Mehran score	1.69	1.88 (1.22-2.91)	0.004
Age	0.005	1.00 (0.97-1.03)	0.750
Sex	-0.061	0.94 (0.47-1.87)	0.861
Contrast volume	-0.001	0.99 (0.99-1.00)	0.341
Contrast type	-0.103	0.90 (0.57-1.42)	0658
∆ Hct	-0.130	0.88 (0.76-1.01)	0.067

BARC, Bleeding Academic Research Consortium; CI, confidence interval; CIN, contrast-induced nephropathy; Hct, hematocrit; LVEF, left ventricular ejection fraction.

## Discussion

In the present study, we examined the incidence of CIN in patients undergoing catheterization procedures via either the TRA or the TFA. We found an overall incidence rate of 12% for CIN in our patients. The incidence of CIN was lower in the TRA approach than in the TFA approach (6.6% vs 15.1%; *P* = 0.01). The volume of the contrast medium used intraprocedurally was higher in the TFA approach than in the TRA approach; nevertheless, the multivariable regression analysis showed that the volume of the contrast medium was not an independent variable for the development of postprocedural CIN (*P* = 0.341). The multivariate analysis showed that the TFA was the independent predictor of the development of CIN (OR: 2.37, 95% CI: 1.11 to 5.10, and *P* = 0.027). Additionally, the BARC and Mehran scores were the other independent predictors of CIN in our study.

The pathogenesis of CIN following diagnostic or interventional coronary procedures is multifactorial. The embolization of cholesterol into the renal arteries during the catheter manipulation in the aorta has been described as a potential mechanism contributing to postprocedural CIN, especially in patients with systemic inflammation, and may be less common in the TRA approach because there is less contact between the catheter and the aortic wall.^21^ Moreover, the TRA is associated with a reduction in vascular complications and bleeding and may, thus, diminish the risk of CIN from hemodynamic instability resulting from hemorrhagic complications.^15,22^

Chiming in with our results, some studies have shown that the TRA may be associated with low rates of CIN by comparison with the TFA.^13-16^ In 2010, British Columbia Cardiac and Renal Registries—the first investigation of its kind to explore the association between the vascular access site and the later onset of a new chronic kidney disease status in patients undergoing cardiac catheterization—showed that the TFA had an OR of 4.36 (95% CI: 2.48 to 7.66) for a new dialysis, a new stage IV or V chronic kidney disease, or a new chronic kidney disease, within 6 months after the cardiac procedure.^16^ Kooiman et al in 2014 matched their study population in terms of propensity and found that the TRA approach was associated with lower adjusted odds of CIN (OR: 0.74 and 95% CI: 0.58 to 0.96) and bleeding (OR: 0.47 and 95% CI: 0.36 to 0.63).^23^ Cortese et al in 2014 showed that the TFA was an independent predictor of CIN (OR: 1.654, 95% CI: 1.084 to 2.524, and *P* = 0.042) in comparison with the TRA in patients undergoing primary PCI.^14^ In a large meta-analysis in 2015, Andò et al found that the TRA approach lowered the incidence of CIN after PCI and this benefit was likely to be mediated by a reduction in bleeding complications.^13^ Recently in 2017, the MATRIX-Access (Minimizing Adverse Haemorrhagic Events by TRansradial Access Site and Systemic Implementation of angioX) trial showed that CIN was 3 times less prevalent and trended lower with the TRA approach (OR: 0.85, 95% CI: 0.70 to 1.03, and *P* = 0.090) than with the TFA approach in patients with the acute coronary syndrome who underwent invasive management. Additionally, post-intervention dialysis was needed in 6 (0.15%) patients treated via the TRA and 14 (0.34%) patients treated via the TFA (*P* = 0.0814).^24^

On the other hand, some studies have found that the choice of the access site is not associated with an increased CIN risk. Damluji et al showed that although the incidence of CIN was low in the TRA approach compared with the TFA approach (2.5% vs 4.5%; *P<*0.001), after adjusting for baseline imbalances, the TFA was no longer associated with an increased risk of CIN.^17^ Kolte et al in a retrospective observational study evaluated the development of CIN in patients with ST-segment elevation myocardial infarction undergoing primary PCI at 2 high-volume tertiary care centers and found no statistically significant association between the choice of the vascular access site and the risk of CIN development.^18^

In conclusion, our study showed that the incidence rate of CIN in patients undergoing diagnostic or interventional coronary management was lower in those treated via the TRA than in the ones treated via the TFA. Furthermore, higher BARC and Mehran risk scores were the independent variables for the development of postprocedural CIN in our patients.

## Competing interests

None to be declared.

## Ethical approval

Informed consent was obtained from all the patients before angiography, and the study protocol was approved by the Review Board of Rajaie Cardiovascular, Medical, and Research Center.
